# Tick populations from endemic and non-endemic areas in Germany show differential susceptibility to TBEV

**DOI:** 10.1038/s41598-020-71920-z

**Published:** 2020-09-23

**Authors:** Katrin Liebig, Mathias Boelke, Domenic Grund, Sabine Schicht, Andrea Springer, Christina Strube, Lidia Chitimia-Dobler, Gerhard Dobler, Klaus Jung, Stefanie Becker

**Affiliations:** 1 Institute for Parasitology, Centre for Infection Medicine, University of Veterinary Medicine Hannover, Hanover, Germany; 2 Research Centre for Emerging Infections and Zoonosis, University of Veterinary Medicine Hannover, Hanover, Germany; 3 Bundeswehr Institute of Microbiology, Neuherbergstraße 11, 80937 Munich, Germany; 4 Institute for Animal Breeding and Genetics, University of Veterinary Medicine Hannover, Hanover, Germany; 5 Parasitology Unit, University of Hohenheim, Stuttgart, Germany; 6 Present Address: Department of Paediatric Pneumology, Allergology and Neonatology, Hannover Medical School, Carl-Neuberg-Str. 1, 30625 Hanover, Germany

**Keywords:** Viral infection, Entomology

## Abstract

Tick-borne encephalitis virus (TBEV) is endemic in twenty-seven European countries, transmitted via the bite of an infected tick. TBEV is the causative agent of one of the most important viral diseases of the central nervous system (CNS). In Germany, 890 human cases were registered between the years 2018–2019. The castor bean tick, *Ixodes ricinus*, is the TBEV vector with the highest importance in Central Europe, including Germany. Despite the nationwide distribution of this tick species, risk areas of TBEV are largely located in Southern Germany. To increase our understanding of TBEV-tick interactions, we collected ticks from different areas within Germany (Haselmühl/Bavaria, Hanover/Lower Saxony) and infected them via an in vitro feeding system. A TBEV isolate was obtained from an endemic focus in Haselmühl. In two experimental series conducted in 2018 and 2019, ticks sampled in Haselmühl (TBEV focus) showed higher artificial feeding rates, as well as higher TBEV infections rates than ticks from the non-endemic area (Hanover). Other than the tick origin, year and month of the infection experiment as well as co-infection with *Borrelia* spp., had a significant impact on TBEV Haselmühl infection rates. Taken together, these findings suggest that a specific adaptation of the tick populations to their respective TBEV virus isolates or vice versa, leads to higher TBEV infection rates in those ticks. Furthermore, co-infection with other tick-borne pathogens such as *Borrelia* spp. can lower TBEV infection rates in specific populations.

## Introduction

Ticks are hematophagous ectoparasites that play a major role in the transmission cycles of various viruses, bacteria, fungi and protozoa^1^. After bacterial borreliosis, tick-borne encephalitis (TBE) is the most important tick-borne disease prevalent in Germany. TBE virus (TBEV), a member of the family *Flaviviridae*^2^, is categorized as an arthropod-borne virus (arbovirus) as it is transmitted by a tick vector. Infection with TBEV can lead to encephalitis in humans^3^ and occasionally animals^4^. Currently, five genetic subtypes of TBEV have been described: the Far Eastern, the Siberian, the European subtype^5^, the recently characterized Baikalian^6^ and Himalayan subtype^7^. The positive-sense single-stranded RNA (+ ssRNA) viral genome encodes for one open reading frame (ORF), which is transcribed to produce a large polyprotein (about 3,400 amino acids in length)^8^. The polyprotein is proteolytically cleaved into three structural proteins (C = capsid, (pr)M = (pre)membrane and E = envelope) and seven non-structural proteins (NS1, NS2A, NS2B, NS3, NS4A, NS4B, NS5)^9^.

TBEV is present in many European countries, from Italy^10^ to Norway^11^. Most recently, the first virus genome sequences were detected in ticks from the UK^12^. In Germany, TBE case numbers have been increasing in the last few years with 890 cases being reported in 2018–2019. A reported 1.5-fold increase in case numbers from a decade earlier (2008–2009; 602 cases)^13^. Anthropogenic as well as environmental parameters are the driving forces for the spread of pathogens and vectors^14^. For example, outdoor activities have increased in recent years, increasing the risk of TBEV transmission to humans. Furthermore, improved diagnostics and enhanced awareness may have increased detection rates of TBEV and contributed to the increase in reported cases. Climate change and higher temperatures may also affect tick behaviour and tick physiology^15,16^ or virus replication rates within infected ticks^17^ and therefore increase the likelihood of TBEV transmission. In contrast to the apparent threat of TBEV infection in the defined risk areas, the vaccination rate in Germany is low. According to recent estimates only 27% of the German population are vaccinated^18^. Therefore, monitoring and evaluation of new and established TBEV endemic foci is necessary to control the risk of TBEV infection in humans. In Germany, *Ixodes (I.) ricinus* is the most important TBEV vector. Despite the nationwide distribution of this tick in Germany, risk areas for TBE are mainly located in the South of the country. *Ixodes ricinus* has a broad host range and thereby complies with properties associated with the ideal vector^19^. Vector competence describes the ability of a given vector to acquire, maintain and transmit a specific pathogen^20^. Analyses of vector competence in hard ticks is specifically challenging due to the complex biological life cycle of these arthropods^21^. Nevertheless, studies that describe tick population and virus strain specific variations of vector infection and transmission efficiency are crucial for risk estimation and public health management. To date, several studies have analysed the interaction of *Ixodes* ticks with *Rickettsia*^22^ and *Borrelia* spp.^23^, but the vector competence of *I. ricinus* populations for TBEV is completely unexplored.

The aim of this current study was to investigate if differences in susceptibility to TBEV infection in different *I. ricinus* populations could explain the unequal distribution of endemic foci in Germany. Therefore, we have adapted a silicone membrane-based artificial feeding system^24^ to infect field-collected *I. ricinus* ticks from one TBEV endemic (Haselmühl, Bavaria) and one non-endemic (Hanover, Lower Saxony) area. The use of field collected ticks for our TBEV infection studies allowed us to correlate factors such as season, year, tick origin and natural co-infection with the infection success of the TBEV offered in our blood meals. We analysed the TBEV infection rates according to month of tick collection in two consecutive years (2018 and 2019) to identify seasonal and annual variation in TBEV infection rates. Furthermore, the collected data were stratified by tick origin and co-infection with the tick-borne pathogens *Borrelia* spp., *Rickettsia* spp. and *Anaplasma *(*A.*)* phagocytophilum*. The data described in this manuscript provide new insights into driving factors for TBEV distribution in nature.

## Results

**Figure 1:**
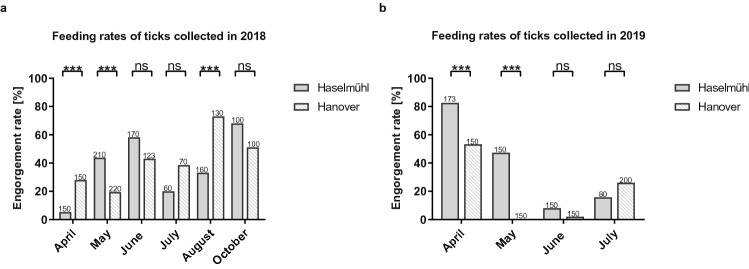
Feeding rates in 2018 (**a**) and 2019 (**b**). Ticks were fed a blood meal containing 1 × 10^6^ PFU of TBEV strain Haselmühl 303/16. The feeding rate (number of engorged ticks divided by the total number of ticks tested) was calculated per month. Data were statistically compared using Chi-square test if all expected counts were 5 or greater, otherwise Fisher’s exact test was chosen using GraphPad Prism 8.3.1. Numbers of ticks tested are indicated above each bar plot. Significant differences are indicated by asterisks (ns; ***p < 0.001).

The susceptibility of German *I. ricinus* ticks to TBEV was analysed using 2,846 nymphs, collected in 2018 in Haselmühl (n = 850) and Hanover (n = 793); and in 2019 in Haselmühl (n = 553) and Hanover (n = 650). Ticks were infected via blood meal and feeding rates (number of engorged ticks divided by the total number of ticks tested) were calculated for every month from April to August and October in 2018 and April to July in 2019 (Fig. 1a,b and Suppl. Table 1). Feeding rates were generally higher in 2018 with mean feeding rates of 40.15% *versus* 29.39% in 2019. Ticks from Haselmühl showed higher feeding activity than ticks from Hanover in May, June and October in 2018 as well as April, May and June in 2019. In contrast, ticks from Hanover showed higher feeding activity than ticks from Haselmühl in April, July and August in 2018 and July in 2019 (Suppl. Table 1).

**Table 1:** Results of binomial GLMs testing the influence of different predictor variables on the probability of TBEV infection after in vitro feeding.

Variable	Estimate	SE	95% CI	*p* value	OR	95% CI
Intercept	2,347	550.9		< 0.0001		
Year (2019 vs. 2018)	− 1.163	0.273	[− 1.710, − 0.639]	< 0.0001	0.313	[0.181, 0.528
Month	− 0.185	0.067	[− 0.319, − 0.056]	0.0057	0.831	[0.727, 0.946]
Tick origin (Haselmühl vs. Hanover)	0.842	0.243	[0.372, 1.326]	0.0005	2.322	[1.450, 3.767]
*Borrelia* spp. infection	0.041	0.361	[− 0.675, 0.745]	0.9107	1.041	[0.509, 2.106]
*Rickettsia* spp. Infection	0.199	0.222	[− 0.237, 0.637]	0.3700	1.221	[0.789, 1.890]
*A. phagocytophilum* infection	0.042	0.348	[− 0.661, 0.714]	0.9044	1.043	[0.516, 2.042]
Interaction tick origin *Borrelia* spp. infection	− 1.045	0.445	[− 1.922, − 0.175]	0.0188	0.352	[0.146, 0.839]

*SE* standard error, *CI* confidence interval, *OR* odds ratio.

Analysis of 693 engorged nymphs from the infection experiments for TBEV RNA revealed that 38.38% (n = 266) of tested ticks were positive. Generally, infection rates in 2019 were lower compared to 2018 (32.33% Haselmühl 2019 versus 52.31% Haselmühl 2018) and the odds for TBEV infection within a tick were 3.3-fold higher in 2018 compared to 2019 (*p* < 0.0001). The month of infection had a weaker impact on TBEV infection with a significant odds ratio (OR) of 0.83 (GLM, Table 1).

**Figure 2:**
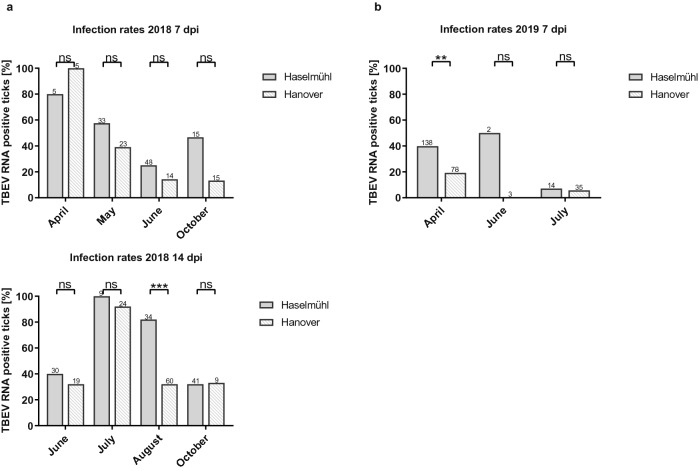
The infection rate (number of positive ticks divided by the number of engorged ticks tested) was calculated in 2018 for 7 and 14 dpi (**a**) and in 2019 for 7 dpi (**b**) over different months. Data were statistically compared using Chi-square test or Fisher’s exact test using GraphPad Prism V8.3.1. Numbers of ticks tested are indicated above each bar plot. Significant differences are indicated by asterisks (ns;* *p* < 0.05; **p < 0.01; ***p < 0.001).

Tick origin also had a significant impact on infection rates. TBEV infection rates in *I. ricinus* nymphs from Haselmühl were generally higher with 2.3 higher odds (*p* = 0.0005) of being infected with TBEV after a blood meal compared to ticks from Hanover (GLM, Table 1). Furthermore, maximum infection rates were higher in Haselmühl nymphs with 80% infection rate at 7 days post infection (dpi) in April in 2018 and 100% infection rate at 14 dpi in July in 2018 (Fig. 2a). However, single month differences were only statistically significant in August in 2018 (14 dpi *p* < 0.0001) and April in 2019 (Fig. 2b, 7 dpi *p* = 5.59 × 10^–3^). *Ixodes ricinus* nymphs from Hanover showed highest infection rates at 7 dpi in April in 2018 (100%, n = 5), which was also the only time we measured higher infection rates in nymphs from Hanover compared to nymphs from Haselmühl. Due to experimental constrains, we can only compare virus loads over time in June in 2018, where we observed a slight increase in infection rates between 7 and 14 dpi (Haselmühl 25% (7 dpi), 40% (14 dpi) and Hanover 14.29% (7 dpi) and 32% (14 dpi)).

**Figure 3:**
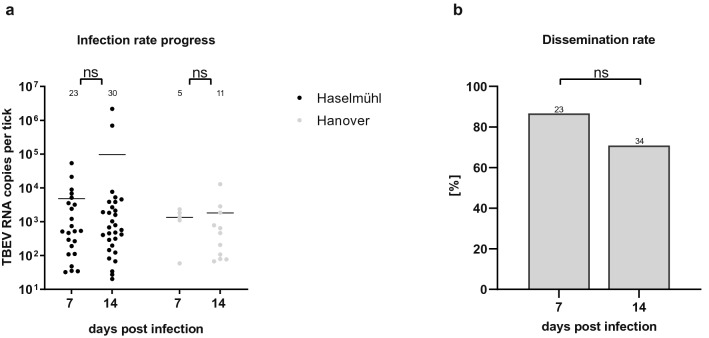
The infection rate progress (**a**) and the dissemination rate (**b**) of ticks in 2018. The gnathosomata and idiosomata from ticks of two populations were dissected and TBEV virus was detected by qPCR. The TBEV-RNA-copy levels were compared among ticks infected in June and October 2018 of different populations at the same time and among ticks of the same populations at different times by mixed-effects analysis using GraphPad Prism V8.3.1. Numbers of ticks tested are indicated above each bar plot. Significant differences are indicated by asterisks (ns *p* ≥ 0.05; *).

To further analyse the viral replication over time, we compared mean RNA copy numbers at 7 and 14 dpi for all experiments (Fig. 3a). We observed a small increase in viral RNA loads in nymphs from Haselmühl with a mean RNA copy number of 4.81 × 10^3^ RNA copies per tick at 7 dpi and 9.72 × 10^4^ RNA copies per tick at 14 dpi. In addition, maximum viral RNA copy numbers increased with time from 5.42 × 10^4^ RNA copies per tick (7 dpi) to 2.18 × 10^6^ RNA copies per tick (14 dpi). In nymphs from Hanover, mean virus RNA copy numbers increased from 1.34 × 10^3^ (7 dpi) to 9.72 10^4^ (14 dpi) RNA copies per tick and maximum viral RNA copy numbers showed similar relations with 2.33 × 10^3^ RNA copies per tick (7 dpi) and 2.18 × 10^6^ RNA copies per tick (14 dpi).

To test if viral RNA copies represent infectious virus, we titrated a subset of our positive samples and measured viral titres between 1.09 × 10^1^ and 1.11 × 10^4^ PFU/tick. Dissemination rates, as a proxy for TBEV circulation in infected ticks, were calculated from the numbers of TBEV-RNA-positive tick´s gnathosomata and idiosomata (Suppl. Figure 1B). A disseminating infection is assumed if an individual tick tests positive for TBEV-RNA in the idosoma TBEV and in the gnatosoma. We observed maximum dissemination rates of 86.67% (total tick number n = 92; TBEV-positive ticks n=23) 7 dpi and lower dissemination rates at 14 dpi (70.83%, total tick number n = 99; TBEV-positive ticks n=34 ) (Fig. 3b).

To analyse the impact of co-infection with other tick-borne pathogens, we tested the engorged nymphs from the infection experiment for DNA from *Borrelia* spp., *A. phagocytophilum*, and *Rickettsia* spp. Out of 640 samples tested, 45% (Haselmühl) and 46% (Hanover) were tested positive for *Borrelia* spp., 7% (Haselmühl) and 3% (Hanover) for *A. phagocytophilum* and 29% (Haselmühl) and 55% (Hanover) for *Rickettsia* spp. in 2018. In 2019, infection with *Borrelia* spp. was observed in 15% (Haselmühl) and 23% (Hanover), infection with *A. phagocytophilum* in 7% (Haselmühl) and 16% (Hanover) and infection with *Rickettsia* spp. in 23% (Haselmühl) and 70% (Hanover) of the ticks tested. The above described infection rates for bacterial pathogens and the TBEV infection rate of 38.38% were used a basis for a correlation analysis of co-infections in our ticks.

Of the three tick-borne pathogens tested, only *Borrelia* spp. infections correlated with TBEV infection rates in *I. ricinus* nymphs when the tick origin was also taken into account. Infection of a tick with *Borrelia* spp. decreased the odds of TBEV co-infection 2.8-fold in nymphs from Haselmühl (Odd ratio: 0.352, *p* = 0.0013; Table 1). *Rickettsia* and *A. phagocytophilum* infection did not show a significant impact on the odds of being infected with TBEV in both tick populations (Table 1).

## Discussion

To gain a more comprehensive understanding of the geographical distribution of TBEV in Germany, we used an artificial feeding system to analyse feeding behaviour and TBEV susceptibility of *I. ricinus* nymphs collected from Bavaria and Lower Saxony. Feeding rates showed high variability over different collection months and the two years of our study. In addition, feeding rates varied between the populations although ticks were sampled in the same period at both sampling sites. Ticks collected within the TBEV focus of Haselmühl showed significantly higher feeding activity in our systems than ticks collected from around Hanover in the months of May and June. In contrast, ticks from Hanover showed significantly higher feeding rates comparatively to Haselmühl in July and August. *Ixodes ricinus* belongs to the so-called exophilic ticks. For these ticks, humidity and temperature play an essential role for host-seeking behaviour^25^. During the feeding experiment, we sought to avoid any variation in temperature, which was constantly kept at 34 °C, and humidity has been kept at 90% RH. However, ticks were collected in the field; hence, all ticks included in our experiments were exposed to environmental conditions beforehand. Temperature and humidity differences in the two sampling locations may have affected the fitness and therefore the feeding success of the field caught arthropods.

By comparing the mean temperatures between Hanover and the town of Amberg, which is the closest weather station to the TBEV focus Haselmühl, we did not find differences throughout the study period, whereas the mean precipitation showed large variations between the two sampling spots as well as between study months and years (Suppl. Table 2). Although we observed higher feeding rates in 6 out of 10 months at the sampling spot with higher precipitation, we could not detect a statistical correlation between these two variables. However, the role of weather conditions during nymphal questing periods and incidence of Lyme disease has been correlated in a US study^26^, indicating a potential link between feeding activity and humidity. Ticks need to leave their sheltered habitat in the ground litter to climb to the tips of grass for host-seeking^27^. This exposure can quickly lead to critical dehydrating conditions for the tick, forcing it to return to the base of the vegetation^28^. Frequent ascending and descending movements can reduce the energy resources of ticks^29^ and lead to a reduction in feeding success.

Besides the different environmental conditions encountered by the arthropods, tick physiological age can influence the feeding acceptance and feeding success of the nymphs. Very little data are available regarding the correlation between physiological age and tick aggressiveness (or feeding success). However, available information on *I. persulcatus* adults indicates increased aggressiveness in physiologically older ticks^30^. Since we collected our ticks via the flagging method, we ensured that nymphs were questing, but the physiological age of the ticks was unknown. Due to diapause, the nymphs collected in April, May and June most probably moulted in autumn of the previous year, whereas ticks collected in July, August and October either moulted in the previous year or were freshly moulted ticks of the respective year^31^. Questing periods of nymphs can extend more than one year, which may have resulted in low energy reserves compromising the feeding success. This might explain the generally lower feeding rates in July of both years despite differing precipitation levels. However, it is striking that ticks collected in the TBEV focus Haselmühl are more active in April-June when most TBEV transmission events occur based on the TBEV case reports^32^. The basis for this high activity of ticks from Haselmühl in April and May are not clear and warrant further investigation. Comparison of the genetic structure of the respective populations regarding energy metabolism might be an exciting first step towards an understanding of population-based differences of feeding activities.

Next to the feeding activity, the ability of ticks to be infected with and to transmit a given pathogen, also known as vector competence, is essential for TBEV transmission cycles. So far, little is known about factors influencing vector competence of an individual tick for TBEV. Previous studies demonstrated vector competence of *Ixodes* ticks for *Borrelia (B) burgdorferi* s.l.^33^, *Rickettsia (R.) monacensis*^22^, and *Powassan virus*^34^. Most extensively, vector competence of different *Ixodes* species for different *B. burgdorferi* s.l. species has been analysed. Those studies have shown remarkable differences in vector competence for *Ixodes* and *Borrelia* depending on population and species^33,35^. Although similar associations of particular tick species, i.e. *I. ricinus* and *I. persulcatus,* with different subtypes of TBEV are known^5^, there is still a major lack of understanding of the specific influences of virus strain, vector species or even population of the same vector species on vector competence for TBEV.

As a first step towards a more comprehensive understanding of TBEV-vector interactions, this study aimed to analyse potential differences in susceptibility to TBEV infection among *I. ricinus* populations from TBEV endemic and non-endemic regions in Germany. Interestingly, we found ticks from the TBEV focus Haselmühl more susceptible to TBEV infection than ticks from the non-endemic area of Hanover. This higher susceptibility cannot be attributed to pre-existing infection with TBEV, since the prevalence of TBEV infection even in endemic foci is only 0.1–5% ^36^. However, it can be argmented that upon the ingestion of a blood meal, physiological processes in the tick can lead to a dramatic increase of endogenous TBEV replication, and thus the before mentioned infection rates of 0.1–5% might be too low. This is supported by data published by Belova et al*.* 2012^37^ and 2017^38^ showing increased TBEV replication after a blood meal. To target this issue, we have conducted a small trial experiment prior to our infection studies with 40 nymphs from Haselmühl and 40 nymphs from Hanover using the same feeding system with non-infected blood and compared TBEV detection rates with questing ticks from the same sampling spots (Suppl. Table 3). We did not detect TBEV RNA in any of the samples analysed, indicating that the blood meal did not have an effect on the TBEV detection probability. Furthermore, field studies conducted by Gerhard Dobler and Lidia Chitimia-Dobler during the past decade showed that the TBEV infection rates at the focus Haselmühl are low with a minimal infection rate of only 0.3% in questing and blood-fed nymphs (1 pool of 6 nymphs out of 410 tested pools containing a total of 991 larvae and nymphs obtained from bank voles was positive for TBEV, Dobler personal communication). Altogether, these data suggest that the infection rates measured in our present study are derived from the TBE virus particles administered in the blood meal and not from pre-existing TBEV infections.

Although the infection differences were rarely statistically significant in single month analysis, except the months August 2018 and April 2019, the overall analysis showed that the odds of becoming infected with TBEV after an artificial blood meal are 2.3 times higher (*p* = 0.0005) for a tick from the TBEV focus Haselmühl. This population-specific difference in the susceptibility to TBEV infection may explain the uneven spread of TBEV in Germany. We hypothesise that the susceptibility for TBEV infection is developed during the co-evolution of a particular TBEV strain and the respective tick population of a natural TBE focus. This assumption is supported by other studies demonstrating the effect of genetic determinants on vector feeding preferences and vector competence. Gerardi et al*.*^39^ analysed genes (16S rRNA and ITS2) of different *Amblyomma sculptum* populations after *R. rickettsii* infection, showing that the two tick populations with the highest susceptibility shared the same 16S rRNA haplotype. Furthermore, genetic adaptation of pathogen and vector has been shown for *I. pacificus* and *I. scapularis* ticks infected with *B. burgdorferi* s.l. The sympatric vector/pathogen pairing showed significantly higher infection rates as compared to the allopatric vector/pathogen pairings^35^. In addition, first evidence of the genetic stability of TBEV strains of particular TBE natural foci over decades demonstrates the optimal adaptation of a TBEV strain to its vector and host in the respective natural focus (Dobler, unpubl. observ and^40^). Interestingly, feeding and transmission rates also differed between the two *Ixodes* species with *I. pacificus* showing higher feeding but lower *Borrelia* transmission rates^35^. For *I. ricinus*, neither the genetic diversity of populations in Germany nor the relationship of genetically distinct populations and vector competence for TBEV has been described so far. More studies are needed to determine if there is genetic diversity amongst tick population in Germany and if so, how the genetic diversity could favour the vector competence for TBEV. Of course, it has to be noted, that the described difference in infection success of TBEV in the tested tick populations does not yet allow conclusions on the respective vector competence of these populations since we have not tested the transstadial transmission of the virus. However, the observed differences will most probably lead to differences in the numbers of infected adults, if transstadial transmission is not significantly different between the tested populations. Further studies are needed to analyse transstadial as well as transovarial transmission of pathogens and the onward transmission to host animals to draw conclusions on differential vector competence of tick populations.

The viral RNA copy numbers found in our study correlate well with the viral RNA copies measured in ticks collected from natural foci (Dobler, unpublished results) and with TBEV RNA copies found in ticks detached from humans, (4 × 10^2^ to 7.7 × 10^6^ TBEV RNA copies/tick^41^). Analysing the TBEV RNA copy numbers over the course of 14 days, we did not observe significant differences between 7 and 14 dpi. In addition, dissemination rates were not significantly different between both time points. However, previous experiments showed that TBEV replication in ticks is enhanced by the blood meal^37^ due to intake of warm blood and changes in biochemical processes in the tick after blood feeding^42^. The only mild increase of viral replication in our study might be attributed to the short incubation time of 14 days in comparison to natural infection, which lasts weeks to months. Furthermore, we infected the tick via blood meal for 5 days, already leading to the above-mentioned changes in biochemical processes triggering the TBEV replication in the process of infection, whereas Belova et al.^37^ infected the tick via intrathoracic injection and offered the blood meal 15 h after initial TBEV infection. In contrast to the observation of Belova et al*.*^37^, Slovak et al.^43^ describe an increase of TBEV replication due to the intake of a blood meal only in injected nymphs but not in nymphs infected via co-feeding, highlighting the role of the infection route for the replication pattern of TBEV. Taken together, our data suggest that TBEV infection after a blood meal is already fully developed as early as 7 dpi.

Besides the genetic adaptation, co-infections are an important influencing factor on tick feeding behaviour as well as on their vector competence. The effects of pathogen infection can either increase or decrease vector survival and fitness. For instance, feeding on *Bartonella*-infected blood decreased the proportion of engorged nymphs and reduced their subsequent weight^44^. Along these lines, the infection with *R. rickettsii* can lead to lethal effects in *Dermacentor (D.) andersoni*^45^. Contrary to this, Lefcort et al*.*^46^ and Hermann et al*.*^47^ could show that *B. burgdorferi* infection promotes host-seeking and nymphal survival under suboptimal environmental conditions. The infection of *I. scapularis* with *A. phagocytophilum* induced the expression of an antifreeze glycoprotein^48^ and heat shock proteins^49^ and *Babesia microti* increases feeding success and survival of *I. trianguliceps*^50^. Further, co-infection can suppress or enhance additional infections. Regarding co-infections in ticks, many studies examine the co-infection status of different bacterial pathogens^51–53^. However, less is known regarding bacterial co-infection and their impact on TBEV infection. This is not surprising since the TBEV prevalence is on a low level with 0.1–5% TBEV-positive ticks in natural endemic foci^36^, making co-infection studies almost impossible. We took advantage of the fact that the nymphs used for in vitro infection experiments originated from natural populations including their natural infection status with other tick-borne pathogens. To maintain the tick microbiota, blood was not supplemented with antibiotics as normally done. After artificial feeding, ticks were tested for their infection status with *Borrelia* spp., *Rickettsia* spp. and *A. phagocytophilum*. TBEV infection rate was 38.38% (n = 693) of overall ticks of 2018 and 2019 making a statistical analysis of co-infection possible.

We did not find a correlation between *Rickettsia* and *A. phagocytophilum* infection and TBEV infection, whereas a *Borrelia* infection lowered the odds of being infected with TBEV. Interestingly, this effect of *Borrelia* co-infection was only apparent for ticks from Haselmühl (OR 0.352; *p* = 0.0188), but not for ticks from Hanover, although the *Borrelia* infection rates were not different between both locations (30.0% Haselmühl and 34.5% Hanover mean values both years). Whether this correlation is biologically relevant and which factors would contribute to a lower TBEV infection rate in *Borrelia-*infected ticks is not clear yet. There might be differences in the composition of *Borrelia* spp. populations from Haselmühl and Hanover, which warrant further investigation. Furthermore, the significance of the here described correlation can only be proved using *Borrelia*/TBEV co-infection models in ticks. Nevertheless, our data provide the first evidence that there might be co-infection interference between *Borrelia* and TBEV. These results would fit well with the observed decrease of *Borrelia* infection rates in Finland^54^, whereas TBEV infection rates show a dramatic increase of TBE in Finland^55^. Additionally, other factors including other microbiota and the virome of ticks could contribute to differences in susceptibility of TBEV between different parts of Germany. Taken together, our data provide evidence that susceptibility for TBEV infection is higher in ticks originating from TBEV endemic foci. This constitutes a reliable database on which further investigations with more virus strains and tick populations of the respective area can be conducted.

## Materials and methods

### Tick sampling and maintenance

Questing *I. ricinus* nymphs were collected in 2018 (April–October) and 2019 (April-July) 3–7 days before artificial feeding by flagging the low vegetation at Hanover (Federal State of Lower Saxony, 52°24′N, 09°51′E) and Haselmühl (Federal state of Bavaria, 49°24′N, 11°52′E). After sampling and feeding, ticks were maintained at room temperature (21 °C) with 95% relative humidity and a 16/8 light/dark photoperiod. During the in vitro feeding, chambers were placed in an incubator with a CO_2_ content of 5%, a relative humidity of about 90% and a temperature of 34 °C. Ticks sampled at the respective area (Haselmühl/Hanover) were fed together in groups of around 50 nymphs per chamber.

### Virus cultivation

The TBEV strain Haselmühl 303/16, was isolated in 2016 from an *I. ricinus* tick pool. A549 cells (ATCC CCL-185) were grown in MEM (Thermo Scientific, Waltham, MA, USA) containing 10% foetal bovine serum (FBS) and antibiotics (penicillin/streptomycin, Pan Biotech; Aidenbach, Germany; gentamicin/amphotericin, Thermo Fisher, Waltham, MA, USA) and maintained at 37 °C under 5% CO_2_ until use. Cells were inoculated with 100 µL aliquots of TBEV-RNA positive tick homogenate provided form the laboratory of the Bundeswehr Institute of Microbiology in Munich, diluted 1:10 in MEM and incubated for 1 h at 37 °C and 5% CO_2_. Unabsorbed virus and potential toxic substances from the tick supernatants were removed by rinsing cells three times with sterile PBS. The infected cells were overlaid with 10 mL of MEM supplemented with 2% FBS and antibiotics (penicillin/streptomycin, Pan Biotech; Aidenbach, Germany; gentamicin/amphotericin Thermo Fisher, Waltham, MA, USA). The virus stock titre was determined by 50% endpoint dilution according to Reed & Muench^56^ and aliquots were stored at − 150 °C. For infection of ticks, the first passage of virus was used.

### In vitro feeding system

The in vitro feeding system was based on the method by Kröber and Guerin^24^, modified substantially in several ways according to safety level of BSL3 (Suppl. Figure 1a). The feeding chamber consisted of two units connected via screw connection. The upper feeding unit was constructed of a glass tube, which was covered with a silicone membrane (bottom side) and a plug for *Drosophila* cultivation tubes 28 × 25 mm (Carl Roth, Karlsruhe, Germany) (upper side). The blood unit consisted of a plastic container (wide-mouth straight-sided PPCO jars with closure, Carl Roth, Karlsruhe, Germany). A seal of two rubber rings prevented leakage of infectious blood and allowed height adjustment of the upper unit to optimise submergence level. For preparation of silicone membranes, lens-cleaning paper (11.6 cm × 7 cm Tiffen, New York, USA) was placed on a transparent cover, which was fixed on a table. Silicone mixture was prepared by mixing 15 g of ELASTOSIL E4 silicone glue (Wacker, Munich, Germany), 4.6 g of silicone oil DC 200 (Sigma-Aldrich, Munich, Germany), 1.3 g of ELASTOSIL colour paste white RAL 9,010 (Wacker, Munich, Germany) with 2.9 g of hexan (Sigma-Aldrich, Munich, Germany). Thin layers of this mixture were spread evenly over the pieces of lens paper by using a metal scraper 80 mm (LUX, Wermelskirchen, Germany). The membranes were left to dry for 48 h at room temperature. After the silicone membranes were dried, glass tube openings were carefully glued with ELASTOSIL E41 RTV-1 (Wacker, Munich, Germany) onto the membranes. The glue was allowed to dry for at least 4 h. Afterwards, the cover was removed carefully with forceps from the membrane. Membranes were examined for leakages by immersion for 30 min in petri dishes filled with 70% ethanol. As olfactory stimulus, animal hair extract was applied to the membrane. Shaved dog hair (Landseer-Golden Retriever-hybrid), was used for the preparation of hair extract. Cut hair was soaked in 350 mL dichloromethane (DCM) for 20 min. Subsequently, 100 mL of DCM was replaced by fresh DCM. This procedure was repeated twice. The mixture was filtered through a Buchner funnel lined with fiberglass filter paper. The tincture was left under the fume hood overnight to evaporate. The hair extract was applied to the silicone membrane and the DCM allowed to evaporate completely. In total, around 50 nymphal *I. ricinus* ticks were added to each feeding chamber. All feeding experiments were conducted in the BSL3** facility of the Research Centre for Emerging Infections and Zoonosis at the University for Veterinary Medicine, Hannover.

### Blood feeding

Sterile, heparinized bovine blood (Fiebig Nährstofftechnik, Idstein, Germany) was used for feeding. Blood was supplemented with 4 g/L D-(+)-glucose monohydrate (Sigma-Aldrich, Munich, Germany), 1 mM adenosine triphosphate as phagostimulant and 3.12 × 10^8^ TCID_50_/mL TBEV isolate Haselmühl 303/16 freshly at each blood change. The final TBEV titre was 3.12 × 10^6^ TCID_50_/mL (1.27 × 10^8^ RNA copies/mL) blood. During artificial feeding, blood was changed twice a day with a maximum time interval of 14 h due to the low stability of TBEV in blood to ensure a constant virus titre (Suppl. Table 4). Ticks were left feeding for 5 days (day -5 to day 0). At day 0, ticks were removed from the membrane, cleaned by immersion in 1% hydrogen peroxide and PBS and transferred to fresh glass tubes for further incubation. At time of collection most ticks were fully engorged. Ticks were then collected for PCR analysis at 7 and 14 days of incubation, further referred to as day 7 and 14 post infection (dpi).

### PCR

Seven and 14 dpi, ticks’ body were cut with a sterile surgical blade to separate gnathosoma from idiosoma (see Suppl. Figure 1b). Single body parts were homogenized in 500 µL cell culture medium (Leibowitz L-15 or MEM-Eagle, Thermo Scientific, Waltham, MA, USA) with 20 Hz, 2 min and 3 repetitions, using steel beads and TissueLyser II (Qiagen, Hilden, Germany). Tick homogenates were clarified by centrifugation, and total RNA was extracted from 140 mL supernatant using the QIAamp Viral RNA Mini Kit (Qiagen, Hilden, Germany) according to the manufacturer’s instructions. Samples were tested for the presence of TBEV RNA by a One-Step quantitative RT-PCR (qRT-PCR) assay using TBEV-specific primers^57^. Standard curve was created using tenfold serial dilutions from TBEV RNA of Austrian Neudoerfl strain (U27495.1), RNase-free water served as a negative control. Each sample was run in duplicate, and the data were analysed using AriaMx software version 1.5 (Agilent Technologies).

For detection of *Borrelia* and *Rickettsia* spp., a duplex quantitative real-time PCR (qPCR) was carried out. *Borrelia* spp. were detected targeting the 5S-23S rRNA intergenic spacer (IGS) region based on a TaqMan minor groove binder (MGB)-probe and primer combination designed by Strube et al.^58^, while the citrate synthase (gltA) gene served as target for amplification of *Rickettsia* spp. using a primer probe combination described by Stenos et al.^59^. For detection of *A. phagocytophilum*, a duplex qPCR targeting the msp2/p44 gene based on a primer–probe combination by Courtney et al.^60^ was performed. Additionally, this duplex qPCR targeted the *I. ricinus* ITS2 region^58^ for species identification and DNA isolation verification.

### Statistical methods

Statistical analyses were conducted in GraphPad Prism V8.3.1. (San Diego, CA ).To assess which factors influenced the likelihood of being infected with TBEV, different models (GLMs) with binomial error structure and logit-link function of logistic regression were fitted. The following predictor variables were included as independent factors: year, month, tick origin, *Borrelia* spp. infection, *Rickettsia* spp. infection, and *A. phagocytophilum* infection. To compare TBEV RNA copy levels among ticks of different populations and at different times, mixed-effect analysis were used. In these models, odds ratios (OR) plus their 95%-confidence intervals were used to quantify the effect of each factor.

Fisher’s exact test and the Chi-square test were applied to assess differences of proportions between the study groups. The significance level was set to alpha = 5%; in cases of multiple testing, *p* values were Šidák-corrected and deemed as statistically significant when corrected values were ≤ 0.05. TBEV RNA copy levels were compared among ticks of different populations and at different times by mixed-effects analysis method.

## Supplementary information

Supplementary information.
