# Supplementary material for: Tick populations from endemic and non-endemic areas in Germany show differential susceptibility to TBEV
Source: Sci Rep. 2020 Sep 23;10:15478. doi: 10.1038/s41598-020-71920-z (PMC7511395; doi:10.1038/s41598-020-71920-z)
Supplement: Supplementary file 1 — Supplementary information. [file 41598_2020_71920_MOESM1_ESM.pdf]

Tick populations from endemic and non-endemic areas in Germany show differential susceptibility to TBEV

Katrin Liebig[1,2], Mathias Boelke[1,2], Domenic Grund[1], Sabine Schicht[1],[5], Andrea Springer[1], Christina Strube[1], Lidia Chitimia-Dobler[3], Gerhard Dobler[3,6], Klaus Jung[4], Stefanie Becker[1,2*]

1 Institute for Parasitology, Centre for Infection Medicine, University of Veterinary Medicine Hannover, Hanover, Germany

2 Research Centre for Emerging Infections and Zoonosis, University of Veterinary Medicine Hanover

3 Bundeswehr Institute of Microbiology, Neuherbergstraße 11, 80937 Munich, Germany

4 Institute for Animal Breeding and Genetics, University of Veterinary Medicine Hannover, Hanover;

5 current address: Department of Paediatric Pneumology, Allergology and Neonatology, Hannover Medical School, Carl-Neuberg-Str. 1, 30625 Hanover, Germany

6 Parasitology Unit, University of Hohenheim, Stuttgart, Germany

* Correspondence should be addressed to Stefanie.becker@tiho-hannover.de

Supplementary Table 1. Feeding rates of *I. ricinus* nymphs after *in-vitro* feeding and infection with TBEV 2018 and 2019.

		2018							
		April		May		June		July	
Tick origin		Feeding rate (%)	p value	Feeding rate (%)	p value	Feeding rate (%)	p value	Feeding rate (%)	p value
Haselmühl		5.33	p< 0.001	43.81	1.84×10^{-6}	58.24	0.9967	20.00	0.1209
Hanover		28.00		19.44		43.01		38.57	
		2018							
		August		October					
Tick origin		Feeding rate (%)	p value	Feeding rate (%)	p value				
Haselmühl		33.13	p< 0.0001	68.04	0.0830				
Hanover		78.08		51.11					
		2019							
		April		May		June		July	
Tick origin		Feeding rate (%)	p value	Feeding rate (%)	p value	Feeding rate (%)	p value	Feeding rate (%)	p value
Haselmühl		82.66	5.20×10^{-8}	47.33	4×10^{-15}	8.00	0.1176	15.83	0.6841
Hanover		53.33		0		2.00		26.00	

Supplementary Figure 1: Tick *In vitro* feeding system adapted to BSL3 conditions and tick dissection

a

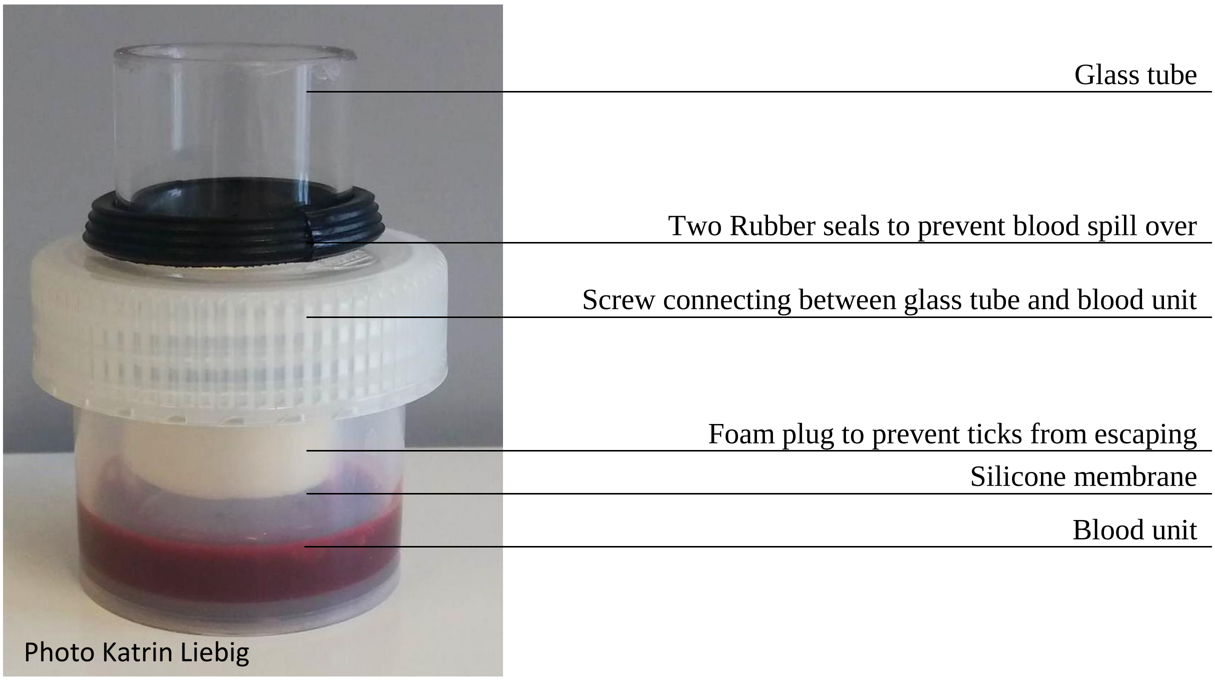

b

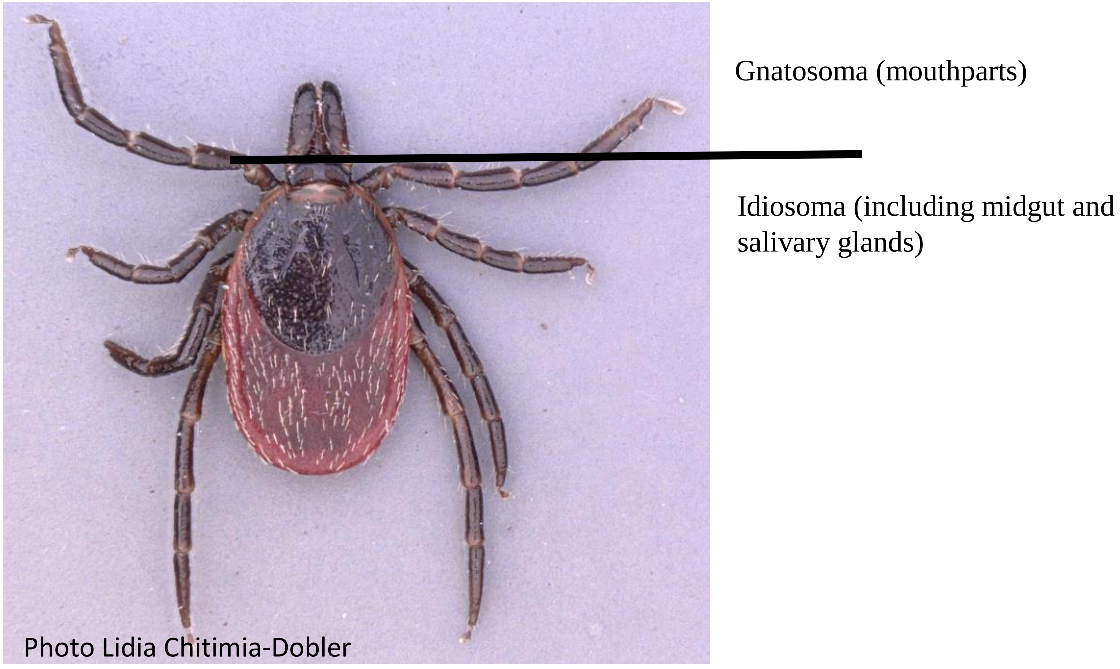

Supplementary Table 2: Mean temperatures and precipitation at the collection sites Hanover and Haselmühl/Amberg in 2018 and 2019 stratified by month and site

Month	Year	Mean temperature (deviation from mean)	Mean temperature (deviation from mean)	Higher feeding/infection rate	Mean precipitation (% of annual mean)	Mean precipitation (% of annual mean)
		Hanover	Amberg		Hanover	Amberg
April	2018	12.6 +3.7	12.9 +4.6	Hanover/ Hanover	26.8 (67%)	14.1 (37%)
	2019	9.9 +1.0	10.0 +1.7	Amberg/ Amberg	26.2 (66%)	19.5 (51%)
May	2018	16.9 +3.5	16.9 +3.6	Amberg/ Amberg	10.0 (18%)	68.2 (108%)
	2019	11.5 -1.9	11.3 -2.0	Amberg/	28.7 (51%)	45.2 (72%)
June	2018	18.1 +2.1	18.6 +2.4	Amberg/ Amberg	21.2 (36%)	32.2 (45%)
	2019	20.3 +4.3	21.0 +4.8	Amberg/ Amberg	42.8 (73%)	37.5 (53%)
July	2018	20.7 +2.3	20.8 +2.5	Hanover/ Amberg	81.8 (134%)	32.0 (41%)
	2019	19.0 +0.6	20.4 +2.1	Hanover/	23.4 (38%)	62.9 (80%)
August	2018	20.0 +2.1	20.8 +3.3	Hanover/ Amberg	38.6 (56%)	45.4 (66%)
October	2018	11.5 +1.6	9.6 +1.1	Amberg/ Amberg	18.4 (35%)	20.2 (37%)

Supplementary Table 3: TBEV detection rates ticks from Hanover and Haselmühl

Ticks were grouped into 5 nymphs per feeding chamber, fed with virus-free blood for 5 days and surviving engorged nymphs were homogenized and tested for TBEV infection using the RT-qPCR assay by Schwaiger *et al.*[1] .

Cohort	No. feeding chamber (number of ticks)	Ticks blood fed (feeding rate)	TBEV detected
Haselmühl	1 (5)	2 (40)	-
	2 (5)	3 (60)	-
	3 (5)	2 (40)	-
	4 (5)	2 (40)	-
	5 (5)	1 (20)	-
	6 (5)	4 (80)	-
	7 (5)	4 (80)	-
	8 (5)	0 (0)	-
**Total Haselmühl**	**40**	**18 (45%)**	**0**
Hanover	9 (5)	2 (40)	-
	10 (5)	0 (0)	-
	11 (5)	4 (80)	-
	12 (5)	0 (0)	-
	13 (5)	5 100)	-
	14 (5)	1 (20)	-
	15 (5)	2 (40)	-
	16 (5)	1 (20)	-
**Total Hanover**	**40**	**15 (37,5)**	**0**

Supplementary Table 4: TBEV viral particles in blood incubated at room temperature for 15h-24h

Sample	TBEV Titre (virus particles)
TBEV spiked blood 0h	$1.61*10^{5}$
TBEV spiked blood 15 h	$1.61*10^{3}$
TBEV spiked blood 24h	$8.18*10^{0}$

References

1 Schwaiger, M. & Cassinotti, P. Development of a quantitative real-time RT-PCR assay with internal control for the laboratory detection of tick borne encephalitis virus (TBEV) RNA. *J Clin Virol* **27**, 136-145, doi:10.1016/s1386-6532(02)00168-3 (2003).
